# Early-onset ventriculomegaly and neuroimmune alterations in *Hoatz*-deficient mice

**DOI:** 10.1186/s12987-026-00796-4

**Published:** 2026-04-01

**Authors:** Keishi Narita, Yoshichika Yoshioka, Sayed Sharif Abdali, Masahiro Ohgidani, Shohei Komaki, Masato Hirakawa, Atsushi Shimizu, Tomoyuki Saino

**Affiliations:** 1 Division of Cell Biology, Department of Anatomy, Iwate Medical University, 1-1-1 Idaidori, Yahaba-cho, Shiwa-gun, Iwate 028-3694 Japan; 2 Division of Ultrahigh Field MRI, Institute for Biomedical Sciences of Iwate Medical University, 1-1-1 Idaidori, Yahaba-cho, Shiwa-gun, Iwate 028-3694 Japan; 3 Department of Functional Anatomy and Neuroscience, Asahikawa Medical University, Midorigaoka higashi, 2-1-1-1, Asahikawa, Hokkaido 078-8510 Japan; 4 Division of Biomedical Information Analysis, Institute for Biomedical Sciences of Iwate Medical University, 1-1-1 Idaidori, Yahaba-cho, Shiwa-gun, Iwate 028-3694 Japan

**Keywords:** Ependymal dysfunction, Hippocampal deformation, T_2_-weighted brain MRI, Ventriculomegaly

## Abstract

Hydrocephalus in mice is frequently associated with impaired motile cilia function. We previously reported that the loss of *Hoatz*, a motile cilia and flagella-associated gene, causes ventriculomegaly of variable severity. Here, we characterize the ventricular phenotype of *Hoatz*^*−/−*^ mice using in vivo magnetic resonance imaging (MRI), histology, and transcriptomics. Severe hydrocephalus occurred in only 4.9% (4/82) of homozygotes. High-resolution MRI revealed a reproducible ~4.7-fold enlargement of the lateral and third ventricles, while overall brain size was preserved. Despite the modest degree of ventriculomegaly, detailed volumetric analyses demonstrated deformation of the surrounding brain parenchyma, most prominently affecting the hippocampus. Ventricular enlargement emerged rapidly during early postnatal development and was occasionally associated with periventricular edema. Morphological and transcriptomic analyses further revealed changes in hippocampal microglia consistent with an altered activation state. Collectively, these findings define *Hoatz* deficiency as a cause of early-onset ventriculomegaly of the lateral and third ventricles, characterized by preserved overall brain size and regional hippocampal deformation, and establish a complementary mouse model for investigating the consequences of altered cerebrospinal fluid dynamics.

**Supplementary information:**

The online version contains supplementary material available at 10.1186/s12987-026-00796-4.

## Background

Ependymal cells are multiciliated epithelial glia that form the lining of the ventricular walls in the central nervous system [1]. These cells are involved in cerebrospinal fluid (CSF) homeostasis, promoting its flow via coordinated beating of motile cilia [2]. Ependymal ciliary abnormalities are frequently associated with severe hydrocephalus in mice, characterized by a dome-like skull resulting from increased intracranial pressure. Several genetic models affecting motile ciliogenesis, including mutations in transcription factor *Foxj1* [3] or axonemal components (e.g., *Spag6* [4] and *Rsph9* [5]), have strongly suggested that impaired ciliary function obstructs CSF flow through narrow passages, such as the cerebral aqueduct, resulting in increased CSF pressure and subsequent ventricular enlargement.

We previously identified *Hoatz* (*Hoatzin*), a vertebrate-specific gene expressed in cells bearing motile cilia and flagella, and demonstrated that *Hoatz* contributes to the structural stability of the ciliary axoneme [6]. Ultrastructural investigation of the ciliary axoneme revealed tissue-specific phenotypes in *Hoatz*-deficient mice, including severe flagellar collapse in late spermatozoa, intermediate structural instability in ependymal cilia, and little to no structural abnormalities in tracheal and oviductal epithelia. Although *Hoatz*-deficient mice develop ventriculomegaly and hydrocephalus, the presentation is milder and relatively more variable than that reported in classical ciliopathy models. However, the onset, severity, and associated structural and cellular changes have yet to be systematically characterized.

To address these gaps, we combined in vivo magnetic resonance imaging (MRI), histological analyses, and transcriptomic profiling to obtain a more detailed characterization of ventricular morphology, brain parenchymal changes, and periventricular cellular responses in *Hoatz*^*−/−*^ mice. These complementary approaches facilitated the establishment of an anatomical and molecular framework for understanding how *Hoatz* loss affects ventricular structure and periventricular tissue integrity. Because our prior ultrastructural work suggested partial instability of ependymal cilia, we hypothesized that such mild dysfunction could drive ventriculomegaly in the absence of the fulminant hydrocephalus typically observed in other ciliopathy models.

## Methods

### Animals

The study protocols were approved by the Institutional Animal Care and Use Committees of Iwate Medical University (approval number: 06–008). All mice were handled according to the Guide for the Care and Use of Laboratory Animals. *Hoatz* mice were maintained on a C57BL/6J background. Heterozygous mutants were bred, and the resulting offspring were genotyped via analysis of genomic DNA extracted from the tail tissue [6]. When wild-type mice were unavailable, heterozygous littermates, which were asymptomatic, served as controls. The animals were housed under a controlled condition with a temperature of 23.5 °C, a humidity level of 50%, and a 12-h day/night cycle. The mice were fed a standard chow diet and provided with water *ad libitum*.

### MRI of the mouse brain under general anesthesia

The mice were anesthetized with 2% isoflurane administered via inhalation for approximately 10 min and then positioned on a small MRI bed equipped with a dedicated mouse brain transceiver microimaging coil [7]. To maintain body temperature at 37 °C ± 0.5 °C and enable continuous monitoring of the respiratory rate, respectively, a heating pad and an air pad, which were connected to a small physiological monitoring system, were placed beneath the mouse’s abdomen. The MRI bed was inserted into a Bruker AVANCE II 11.7T ultra-high-field NMR/MRI system operated using the ParaVision 6.0 software (Bruker BioSpin, Ettlingen, Germany).

T_2_-weighted brain images were acquired using a turbo rapid acquisition with relaxation enhancement (T_2_-Turbo RARE) sequence and the following key parameters: echo time, 30.96 ms; repetition time, 4000 ms; number of signal averages, 2; RARE factor, 4; slice thickness, 0.5 mm with no interslice gap; matrix size, 512 × 512 pixels; and field of view, 20 × 20 mm. Image stacks covering nearly the entire brain were acquired in three mutually orthogonal planes, namely, coronal (33 slices), horizontal (15 slices), and sagittal (21 slices) planes. The total imaging time per mouse was approximately 50 min. All images were stored in the DICOM format. After imaging, the mice were returned to their home cages and closely monitored until full recovery from anesthesia.

### Measurements of ventricular volume and brain size using serial MRI slice images

For ventricular volume quantification, Fiji (ImageJ v1.54f; National Institutes of Health, USA) [8] was used for image segmentation as follows: Serial 32-bit DICOM images were imported as an image sequence, and the brightness/contrast were reset. Then, the image dimensions (pixel width and height: 0.0390625 mm; voxel depth: 0.4966705 mm) were confirmed using the Image > Properties menu. Ventricular regions were segmented using the Wand tool set to four-connected mode and a tolerance of 1. Subsequently, binary mask images were generated for each slice, saved using numerically sorted filenames, and then reimported as a new image sequence. The lookup table was configured to render masks in white against a black background. A threshold was applied, and the “Analyze Particles” function was utilized with default parameters (size, 0–infinity; circularity, 0.00–1.00; show, nothing; “Clear results” and “Summarize” options enabled) to measure the area of the ventricular masks in each slice.

The total ventricular volume was calculated by summing the cross-sectional areas across all slices and multiplying by the voxel depth. The final volume for each mouse was calculated as the mean of measurements obtained from the three orthogonal image sets. The 3D Viewer plugin in Fiji [9] was used to generate a 3D Z-stack visualization of the ventricular structure, with the resampling factor set to 1.

For brain size measurements, three mutually orthogonal DICOM datasets (coronal, sagittal, and horizontal) from each mouse were loaded into the 3D Slicer software (v5.6.2) [10] to generate a four-up view. To measure brain dimensions—left–right, dorsal-ventral, and rostral–caudal lengths—straight lines were drawn along the intersections of three reference planes using the Line tool. These reference planes were defined based on the following anatomical landmarks: the midline sagittal plane encompassing the third ventricle, the horizontal plane encompassing the foramina of Monro, and the coronal plane encompassing the median eminence at the infundibular recess of the third ventricle. The rostral endpoint was defined as the boundary between the olfactory bulb and cerebral hemispheres, whereas the caudal endpoint corresponded to the posterior margin of the cerebral hemispheres.

To measure total brain volume in each MRI slice, the brain outline was manually traced using the freehand selection tool in Fiji/ImageJ on a Wacom DTK2241 tablet (Wacom, Saitama, Japan). The brain parenchyma volume was then calculated by subtracting the measured ventricular areas.

Statistical analysis and data visualization were performed using GraphPad Prism software (v8.4.3; GraphPad Software, San Diego, CA, USA). Data were presented as scatter dot plots, with each dot corresponding to a single mouse and horizontal bars denoting group means. Comparisons between two groups were performed using Mann–Whitney U test, and *p*-values < 0.05 were considered statistically significant. For slice-by-slice comparison of total brain and parenchyma volume, the results were presented as mean ± standard deviation. A mixed-effects model was applied for statistical testing, with slice position as the within-subject factor and genotype as the between-subject factor, using the Geisser–Greenhouse correction. Sidak multiple comparison test was used as a post hoc procedure to evaluate pairwise differences for each slice. The effect sizes for these pairwise comparisons were estimated using Cohen *d* [11] to obtain an index of the magnitude of genotype-related differences.

### Immunostaining for Iba1 and fluorescence microscopy

For immunohistochemical analysis, mice were euthanized at the age of 1.5 weeks (postnatal days 10–11) via intraperitoneal injection of a triple-combination anesthetic (0.75 mg/kg of medetomidine, 4.0 mg/kg of midazolam, and 5.0 mg/kg butorphanol in distilled water), followed by transcardial perfusion with 4% (w/v) paraformaldehyde in phosphate-buffered saline (PBS) at a volume > 1 mL/g body weight. Subsequently, brains were dissected and post-fixed in the same fixative for 24 h at 4 °C. Next, tissues were cryoprotected in 30% (w/v) sucrose in PBS until equilibration and then embedded in Tissue-Tek® O.C.T. Compound (Sakura Finetek, Tokyo, Japan). Coronal brain sections (50-μm thick) were prepared using a Leica CM3050S cryostat, and sectioning coordinates were determined according to the Allen adult mouse brain reference atlas (Allen Institute for Brain Science), targeting the hippocampal region corresponding to approximately image 66 of 132 in the Interactive Atlas Viewer (https://atlas.brain-map.org/) [12]. Afterward, the sections were transferred to a multi-well plate containing PBS to dissolve the embedding medium.

The floating sections were permeabilized with 0.3% (v/v) Triton X-100 in PBS overnight at 4 °C. After permeabilization, the sections were blocked for 3 h at room temperature in PBS containing 10% (v/v) normal donkey serum and 0.1% (v/v) Triton X-100. Then, the sections were incubated with rabbit anti-Iba1 polyclonal antibodies (#019–19741, 1:1,000, Fujifilm Wako, Tokyo, Japan) diluted in blocking buffer for 48 h at 4 °C. After being washed with PBS containing 0.1% (v/v) Tween 20 (PBST), the sections were incubated with a mixture of Cy3 AffiniPure donkey anti-rabbit IgG antibodies (#AB_2307443, 1:1,000, Jackson Laboratories, USA) and DAPI nuclear stain (#340–07971, 1 μg/mL, Dojindo, Kumamoto, Japan) overnight at 4 °C. After being washed with PBST, the brain sections were transferred onto a gelatin-coated glass slide, briefly dried for 10 min at 37 °C, and then sealed with an aqueous Fluoroshield mounting medium with DAPI (#AB_104139, Abcam, USA).

Fluorescence microscopy was performed using a Nikon Eclipse Ti2 inverted microscope system equipped with a Nikon A1R laser confocal microscope scanning unit (Nikon, Tokyo, Japan). Imaging was performed at room temperature using a Plan Apochromat Lambda 100× oil immersion objective (NA 1.45, WD 0.17). The samples were excited with 404- and 560-nm laser lines for DAPI and Cy3, respectively, each at 2% laser power. Emission signals were collected using appropriate bandpass filters (452/45 and 593/46 nm for DAPI and Cy3, respectively) and detected using GaAsP photomultiplier tubes (PMT HV: 140 for DAPI, 77 for Cy3; offset: 0). Confocal images were sequentially acquired in the Galvano scanning mode using a large field of view head (FOV25) to avoid spectral crosstalk. Scans were performed at a resolution of 1024 × 1024 pixels with no line averaging and a scanner zoom of 0.72 × . The scan speed was set to 0.125 (one-way scan direction), and the pinhole size was 47.25 μm, corresponding to approximately 1.2 Airy units. Z-stacks were captured using Ti2 ZDrive across 80 optical sections with a step size of 0.5 μm.

Images were acquired and managed using the NIS-Elements AR software (Nikon, Tokyo, Japan), and postprocessing, including noise reduction, was performed using the Denoise.ai function. In addition, volume rendering of confocal stacks was performed using the NIS-Elements AR software in the depth-coded blending mode.

After acquisition, the images were reconstructed and rendered into 3D microglial models using the Imaris 9.6 software (Bitplane, Zurich, Switzerland). Morphological parameters, including cell surface area and cell volume, were measured using the Imaris MeasurementPro module. Then, the microglial ramification ratio (surface area/volume) was calculated, as previously described [13, 14]. A total of 275–327 microglia per group were analyzed, obtained from four sections per mouse (*n* = 3 mice per group). Statistical analysis was conducted using the unpaired, two-tailed *t*-test for comparisons between two groups, and *p* < 0.05 was considered to indicate statistical significance. Data were visualized using violin plots.

### Hippocampal RNA extraction and transcriptome analysis

Three pairs of male littermate mice (wild-type and *Hoatz*^−/−^) were euthanized via cervical dislocation at 15, 21, and 25 weeks of age (postnatal days 108, 146, and 172). For total RNA extraction, hippocampal tissues were rapidly dissected in ice-cold HBSS followed by homogenization in ice-cold TRIzol reagent (Thermo Fisher Scientific, USA), according to the manufacturer’s protocol. RNA pellets were dissolved in DEPC-treated water, and the RNA concentrations were measured using a NanoDrop spectrophotometer (Thermo Fisher Scientific, USA). The samples were stored at −80 °C until further processing.

Genome-Lead Inc. (Kagawa, Japan) performed RNA quality assessment, mRNA library preparation, and RNA sequencing. Briefly, total RNA was processed using the KAPA mRNA Capture Kit (KAPA BIOSYSTEMS, Inc., USA) for poly(A) RNA enrichment. Furthermore, strand-specific libraries were prepared using the MGIEasy RNA Directional Library Prep Set (MGI Tech Co., Ltd., China). The libraries were sequenced on the DNBSEQ-T7RS platform (MGI) using 150-bp paired-end reads. The resulting data were analyzed as follows: First, the quality of raw sequencing reads (FASTQ files) was assessed using FastQC (v0.12.1). Second, adapter sequences and low-quality bases were removed using Trimmomatic (v0.39) with default settings. Third, the resulting reads were mapped to the mouse reference genome (GRCm39, excluding ribosomal RNA sequences) using STAR (v2.7.10b). Fourth, gene expression levels were quantified using RSEM (v1.3.1), generating expected read counts and TPM values. Fifth, the counts per million (CPM) were calculated from the raw count data using the cpm() function in the edgeR package. Sixth, principal component analysis (PCA) was conducted using the prcomp() function in R with the option scale = TRUE, based on the log-transformed CPM values. Seventh, differential gene expression analysis was conducted in R using the edgeR package (v3.34.1) and its glmLRT() function, and genes with a false discovery rate–adjusted *p*-value < 0.05 and an absolute log_2_ fold change (|log_2_FC|) ≥2 were considered to be significantly differentially expressed. To identify significantly enriched biological processes and pathways, gene set enrichment analysis was conducted using the Metascape online platform (v3.5).

## Results

### Low incidence of severe hydrocephalus in homozygous *Hoatz* mutants

We previously observed hydrocephalus with variable severity in homozygous *Hoatz* mutants [6]. To quantitatively assess this variability, we monitored the incidence of severe hydrocephalus while maintaining the colony by crossing heterozygotes. Heterozygous intercrosses produced 271 pups (females: 137, males: 134) during the past year, with a genotype distribution of 55 wild-type (+/+), 134 heterozygous (+/−), and 82 homozygous mutant (−/−) offspring (Table 1). The proportion of wild-type pups (20.3%) was slightly lower than the Mendelian expectation (25%), whereas the proportion of homozygous mutants was moderately higher (30.3%). No statistically significant sex bias was observed in any genotype group.

**Table 1:** Distribution of offspring genotypes, sex, and incidence of growth retardation associated with a domed skull in 271 pups obtained from 35 heterozygous intercrosses over the past year

*Hoatz* genotype	Sex (female/male)	Growth retardation and domed skull, n (%)
+/+	55 (24/31)	0 (0%)
+/*−*	134 (74/60)	0 (0%)
*−*/*−*	82 (39/43)	4 (4/0; 4.9%)

Values are shown as number (n) and percentage (%). Female and male counts are indicated in parentheses. Growth retardation associated with a domed skull was assessed on postnatal day 21.

Abnormal postnatal deaths or euthanasia due to marked growth retardation associated with a domed skull were recorded in four animals, all of which were homozygous females (4/82, 4.9%). To investigate whether these deaths reflected an underlying systemic defect, we assessed the cardiac function of *Hoatz*^*−/−*^ mice by echocardiography, but no significant abnormalities were detected (Drs. M. Nishida, A. Nishimura, and X. Tang; National Institute for Physiological Sciences; personal communication). Thus, the homozygous mutants were considered essentially viable under the current breeding conditions, with a low incidence of severe hydrocephalus. No deaths were observed among wild-type or heterozygous littermates.

### MRI of the brain ventricles in wild-type and *Hoatz* knockout mice

We used high-resolution T_2_-weighted MRI for in vivo examination of ventricular morphology in six pairs of adult male littermates (wild-type and *Hoatz*^*−/−*^, aged 6–10 weeks; postnatal days 40, 50, 50, 53, 65, and 74). Each mouse was anesthetized using gas, secured on an MRI bed, and placed into the imaging system. After aligning the three mutually orthogonal planes, three datasets were acquired, comprising 69 images (33 coronal, 15 horizontal, and 21 sagittal stacks). The key imaging parameters were as follows: field of view, 20 × 20 mm; in-plane resolution, 512 × 512 pixels; and slice thickness, 0.5 mm with no interslice gap. Imaging was performed using a T_2_-Turbo rapid acquisition with relaxation enhancement (RARE) sequence and completed within 50 min. All mice fully recovered from anesthesia after imaging.

**Fig. 1:**
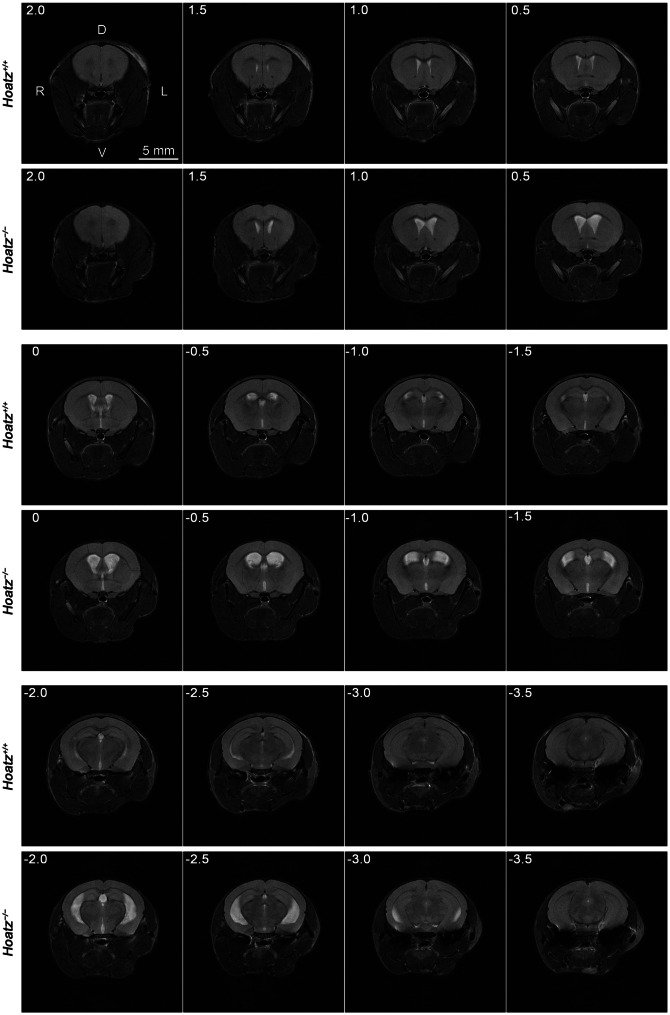
Representative montage of serial T_2_-weighted MRI sections of 6-week-old *Hoatz*^*+/+*^ and *Hoatz*^*−/−*^ mice. The slice dimensions were 20 × 20 mm, and the thickness was 0.5 mm with no interslice gaps. Sections are arranged from the rostral to the caudal direction. The number in the upper left corner of each section indicates the distance (mm) from the anterior commissure. The skin indentation at the lower right of the head was caused by the gas anesthesia tubing

The acquired image stacks nearly covered the entire brain. Although the protocol offered high contrast and resolution, with clear visualization of CSF-filled ventricular spaces as hyperintense regions (Fig. 1), the cerebral aqueduct (the narrow passage connecting the third and fourth ventricles) was not clearly detectable. Blood vessels appeared as hypointense tubular structures. Marked lateral ventricular enlargement was consistently observed in all six *Hoatz*^*−/−*^ mice and appeared to be accompanied by thinning of the surrounding brain parenchyma, particularly in the dorsal hippocampal region, as subsequently quantified. However, the overall brain size remained relatively unchanged (Fig. 1). Edema-like hyperintense lacunae were observed within the corpus callosum of one *Hoatz*^*−/−*^ mouse (Supplementary Figure S1).

To further support the MRI findings, we performed a preliminary histological assessment using brain tissue samples from two heterozygous and two homozygous adult males. The tissue was fixed in formalin, embedded in paraffin blocks, and sectioned coronally (10 µm thick at 0.1 mm intervals). Following staining using the Klüver–Barrera method, they were examined under light microscopy. The histological observations were consistent with the MRI findings, further supporting the significant lateral ventricular expansion in *Hoatz*^*−/−*^ mice (Supplementary Figure S2). Edematous spaces were also observed in the corpus callosum and periventricular white matter in one homozygote. Consistent with our previous report [6], heterozygotes were asymptomatic, with no detectable ventricular expansion. Collectively, these findings show that *Hoatz*^*−/−*^ mice exhibit mild yet reproducible ventriculomegaly, occasionally accompanied by lacunar lesions in periventricular white matter.

### Measurements of ventricular volume and brain size using serial MRI slice images

**Fig. 2:**
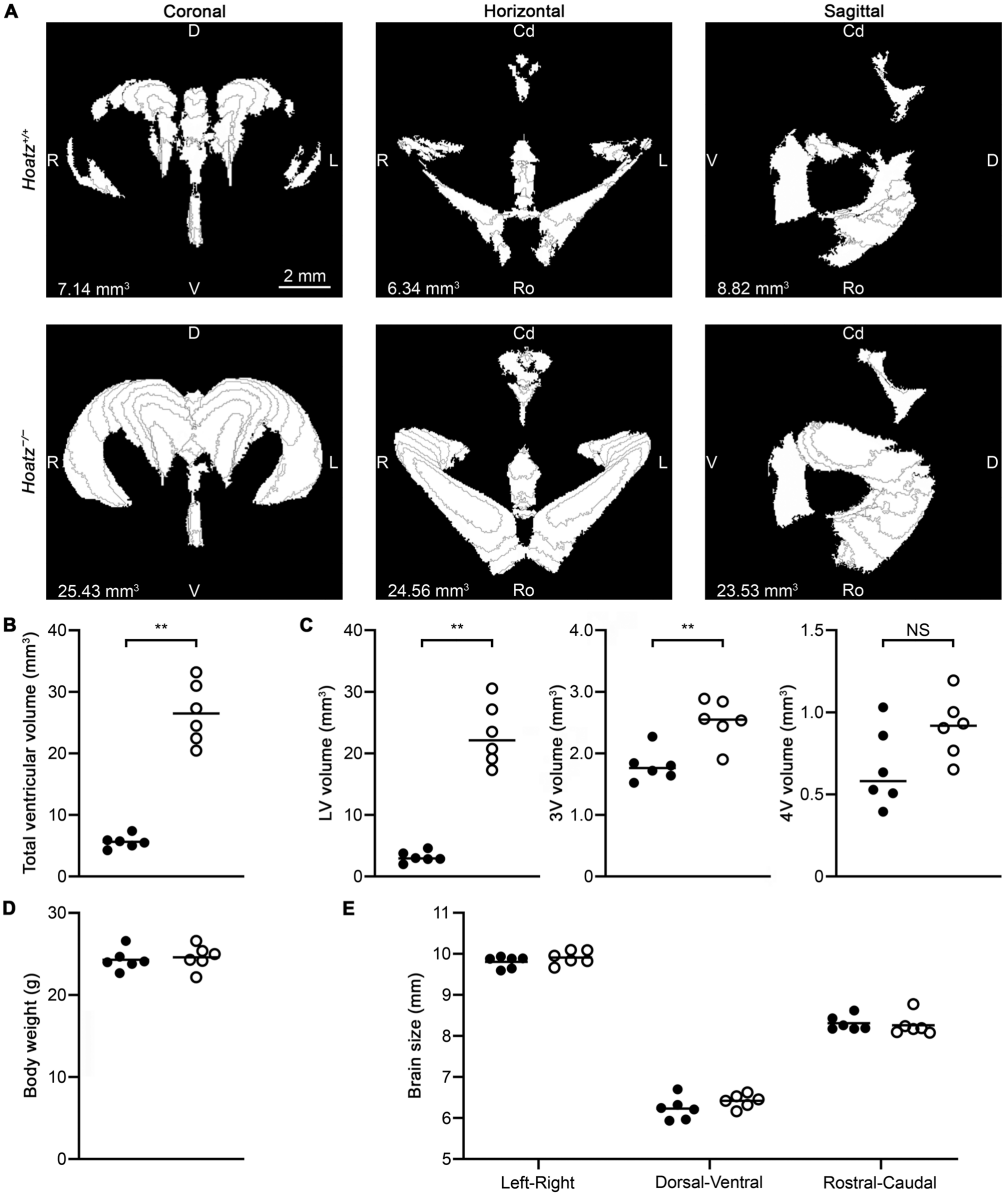
Measurements of ventricular volume and brain size. (**A**) Representative segmentation and volume measurement of the brain ventricles of 6-week-old wild-type and null mice. The hyperintense areas corresponding to ventricular lumina were selected using the wand tool in Fiji/Imagej to create masks. The ventricular volumes were estimated from each of the three mutually orthogonal image stacks and then averaged. (**B**–**E**) Filled circles, wild-type; open circles, null. (**B**) Comparison of total ventricular volume (scatter dot plots with mean bars). **, *p* = 0.0022, Mann–Whitney U test, *n* = 6. (**C**) The data presented in (**B**) were further subdivided to separately analyze the lateral, third, and fourth ventricles. **, *p* < 0.005, Mann–Whitney U test; NS, not significant. (**D**) Comparison of body weight. (**E**) Comparison of brain size

We measured the ventricular volume and overall brain size using serial MRI slice images and comparing these values between wild-type and *Hoatz*^*−/−*^ mice. First, ventricular volumes were measured using the Fiji/ImageJ software, by selecting hyperintense regions corresponding to the ventricular lumina using the wand tool and converting them into binary masks (Fig. 2A). Then, ventricular volume was calculated by counting the total number of pixels within the masks across slices. Because the measurements from three mutually orthogonal image stacks were consistent across animals (mean coefficient of variation: 8.3%, range: 1.0%–17.5%, *n* = 12), the average of the three stacks was considered the representative ventricular volume for each mouse. The average ventricular volume of *Hoatz*^*−/−*^ mice (26.51 ± 4.95 mm^3^, mean ± SD) was ~4.7-fold greater than that of wild-type mice (5.66 ± 1.05 mm^3^, *p* = 0.0022, Mann–Whitney U test, *n* = 6) (Fig. 2B). Separate quantification of the lateral, third, and fourth ventricles revealed marked enlargement of the lateral and third ventricles in *Hoatz*^*−/−*^ mice compared with wild-type mice (Fig. 2C).

The body weights measured on the day of imaging were comparable between the two genotypes (Fig. 2D). The overall brain sizes—left–right, ventral–dorsal, and rostral–caudal lengths of the cerebrum—measured using 3D Slicer software did not differ significantly between the two genotypes (Fig. 2E).

### Measurements of brain volume and calculation of parenchymal volume

**Fig. 3:**
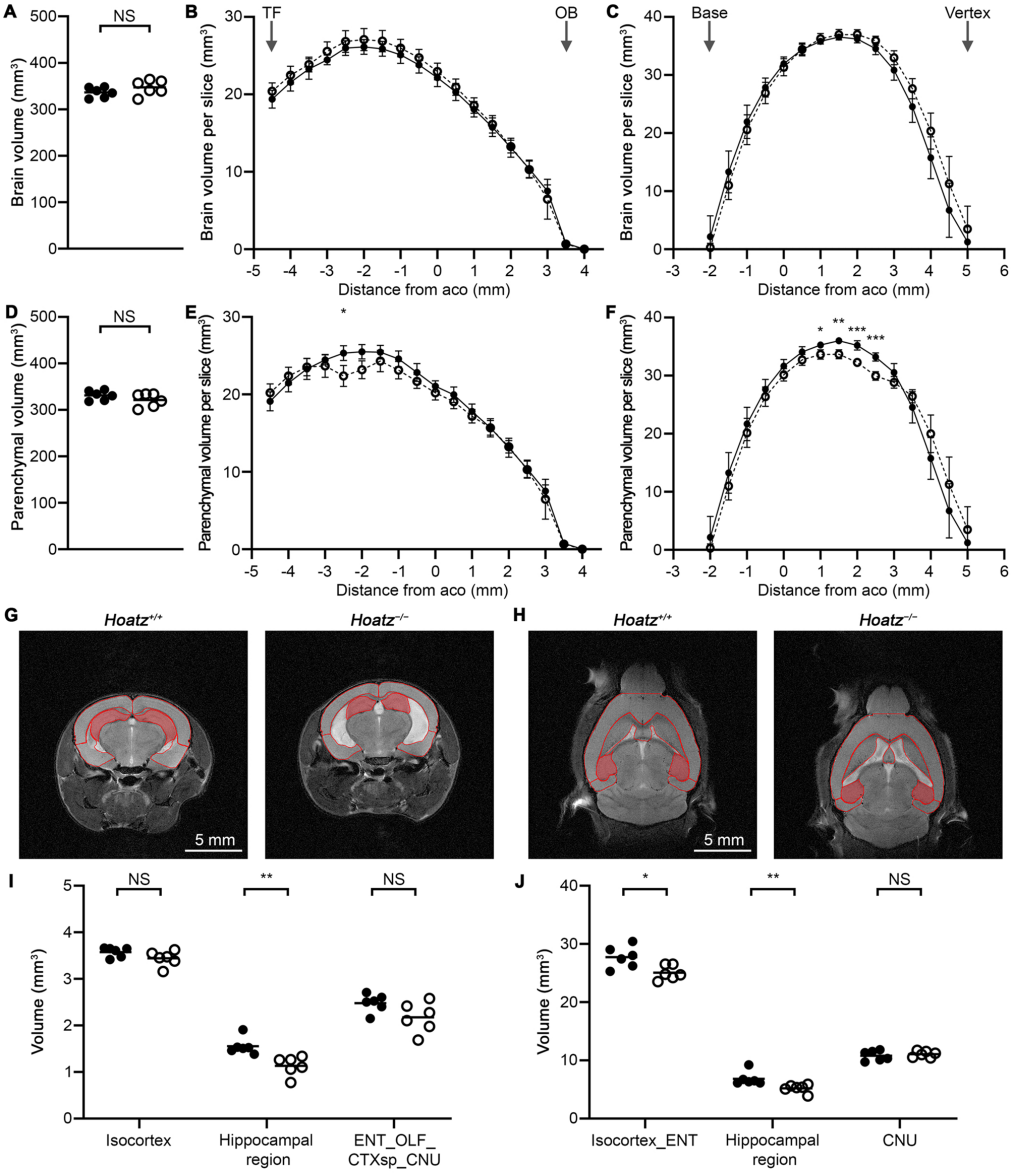
Brain and parenchymal volume measurements. (**A**–**F**, **I**, **J**) Filled circles, wild-type; open circles, null. (**A**) Comparison of total brain volume (scatter dot plots with mean bars). (**B**) Slice-by-slice comparison of brain volume using coronal sections (mean ± standard deviation). TF, transverse fissure; OB, olfactory bulb. (**C**) Slice-by-slice comparison of brain volume using horizontal sections (mean ± standard deviation). (**D**) Comparison of total parenchymal volume. (**E**) Slice-by-slice comparison of parenchymal volume using coronal sections. *, *p* = 0.0316 (Sidak multiple comparison test), *n* = 6. (**F**) Slice-by-slice comparison of parenchymal volume using horizontal sections. *, *p* = 0.0402, **, *p* = 0.0029, ***, *p* < 0.0007 (Sidak multiple comparison test), *n* = 6. (**G**) Representative coronal slice at −2.5 mm from the anterior commissure. The cerebrum was segmented into three regions, and the hippocampal regions are highlighted. (**H**) Representative horizontal slice at 2 mm from the anterior commissure. The cerebrum was segmented into three regions, and the hippocampal regions are highlighted. (**I**) Comparison of regional parenchymal volumes in the coronal slice at −2.5 mm from the anterior commissure. ENT, entorhinal area; OLF, olfactory areas; CTXsp, cortical subplate; CNU, cerebral nuclei. **, *p* = 0.0022, Mann–Whitney U test, *n* = 6. (**J**) Comparison of the regional parenchymal volumes in the horizontal slices at 1.0–2.5 mm from the anterior commissure. *, *p* = 0.0238, **, *p* = 0.0022, Mann–Whitney U test, *n* = 6

We also quantified total brain volume in MRI sections containing the cerebrum. First, we manually traced the brain outline in each section using the freehand selection tool in Fiji/ImageJ. Then, we multiplied the traced area by the slice thickness to obtain the volume. For slice-by-slice volume comparisons, the anterior commissure was used as an anatomical landmark for aligning the rostro-caudal and dorso-ventral positions. Consistent with the brain size measurements described above, there were no statistically significant differences in total brain volume between wild-type and *Hoatz*^*−/−*^ mice (336.6 ± 11.1 vs. 347.4 ± 16.1 mm^3^, mean ± SD; Fig. 3A). Slice-by-slice analysis revealed a trend toward increased volume in *Hoatz*^*−/−*^ mice across the coronal, horizontal, and sagittal datasets, but this was not statistically significant (Fig. 3B, C and Supplementary Fig. S3A, B).

Brain parenchymal volume was calculated by subtracting the aforementioned ventricular volume from the total brain volume. Overall parenchymal volume was similar between wild-type and null genotypes, with no significant differences (331.5 ± 10.4 vs. 321.7 ± 14.4 mm^3^, mean ± SD; Fig. 3D). In contrast, slice-by-slice comparisons revealed a trend toward reduced parenchymal volume in *Hoatz*^*−/−*^ mice, with several slices exhibiting statistically significant reductions (Fig. 3E, F and Supplementary Fig. S3C, D). The level of sensitivity for detecting genotype-dependent differences varied across the three orthogonal datasets. The horizontal dataset provided the highest statistical power, as evidenced by the plot of effect sizes (Supplementary Fig. S3E).

To further investigate the cerebral regions contributing to the observed differences, we focused on slices showing significantly reduced parenchymal volume in the coronal and horizontal datasets. Segmentation of cerebral parenchyma within these regions revealed significant reduction in hippocampal volume in *Hoatz*^*−/−*^ mice. In the coronal dataset, hippocampal volume at −2.5 mm from the anterior commissure was reduced by approximately 27% compared with wild-type controls (1.56 ± 0.18 mm^3^ vs. 1.14 ± 0.21 mm^3^, mean ± SD; *p* = 0.0022, Mann–Whitney U test, *n* = 6). Similarly, in the horizontal dataset, hippocampal volume integrated across slices spanning 1.0–2.5 mm from the anterior commissure was decreased by approximately 24% in *Hoatz*^*−/−*^ mice (6.86 ± 1.19 mm^3^ vs. 5.22 ± 0.70 mm^3^, mean ± SD; *p* = 0.0022, Mann–Whitney U test, *n* = 6) (Fig. 3G–J). Additionally, the horizontal datasets showed a reduction (~10%) in the iso- and entorhinal cortices (27.76 ± 1.87 mm^3^ vs. 25.07 ± 1.23 mm^3^, mean ± SD; *p* = 0.0238, Mann–Whitney U test, *n* = 6). These findings indicate that ventriculomegaly in *Hoatz*^*−/−*^ mice is accompanied by deformation of the surrounding brain parenchyma, mainly in the hippocampus.

### Onset of ventriculomegaly during weaning and detection of hippocampal microglial activation

**Fig. 4:**
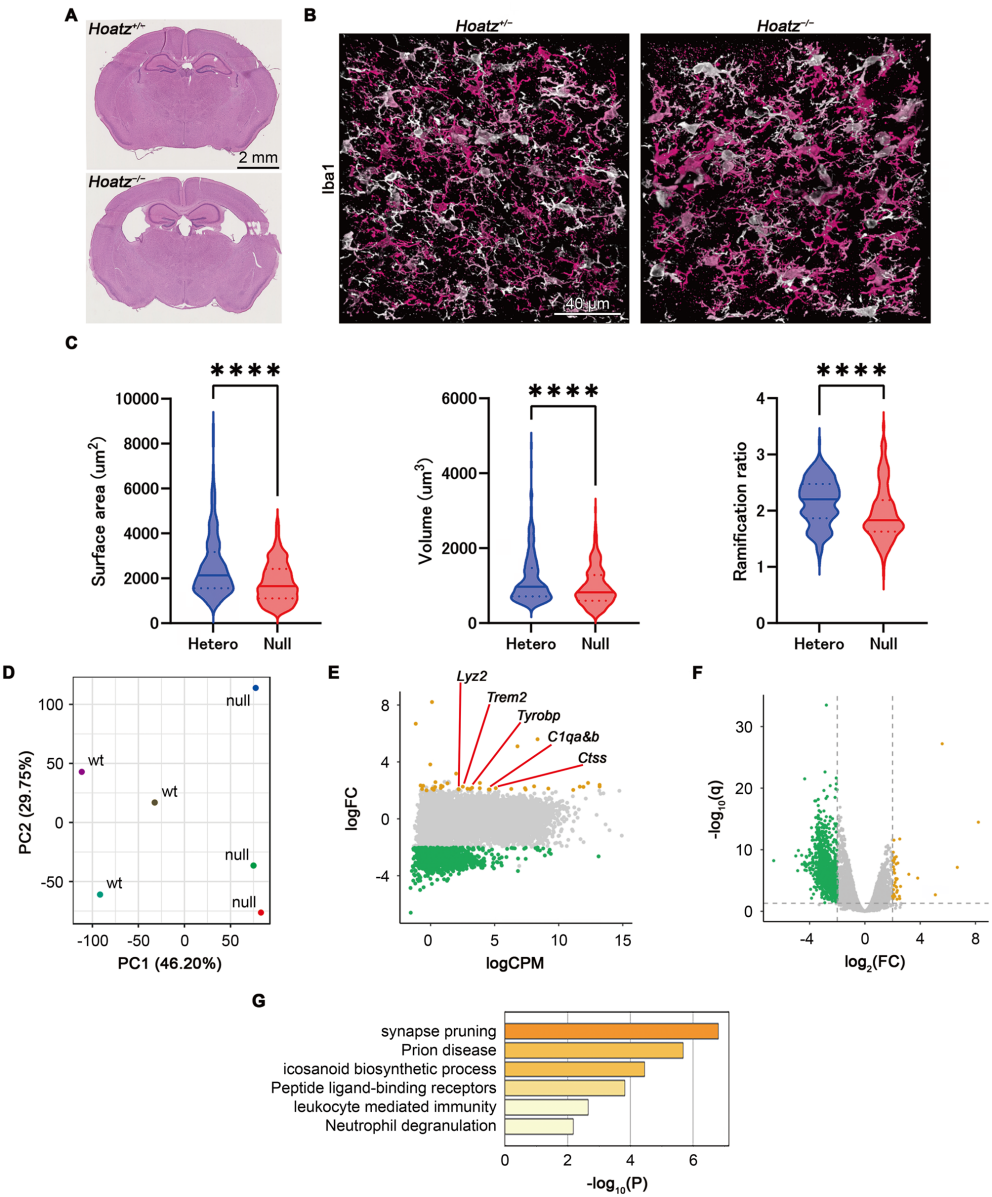
Detection of ventriculomegaly at the weaning stage and evidence of hippocampal microglial activation. (**A**) Representative hematoxylin and eosin-stained coronal brain sections at 1.5 weeks of age. (**B**) Representative 3D confocal image of Iba1-positive microglia in the hippocampus (174 × 174 × 40 μm) at 1.5 weeks of age, shown with depth color coding (lighter colors indicate more superficial optical planes and darker pink colors indicate deeper planes). (**C**) Morphological analysis of hippocampal microglia. Violin plots show the surface area, volume, and ramification index in asymptomatic heterozygous and *Hoatz*^*−/−*^ mice. ****, *p* < 0.0001, two-tailed *t*-test, *n* = 275–327. (**D**) Principal component analysis of RNA sequencing data. (**E**) MA plot showing microglia-related genes upregulated in the hippocampus of *Hoatz*^*−/−*^ adult mice. Orange, upregulated genes; green, downregulated genes. (**F**) Volcano plot of differentially expressed genes (adjusted *p* < 0.05, |log2 Fold change| ≥2). Orange, upregulated genes; green, downregulated genes. (**G**) Gene ontology enrichment analysis of upregulated genes

To determine when ventricular enlargement emerges, we performed histological examination of formalin-fixed sections of brain tissue harvested from mice at birth and at 1.5 weeks of age. Hematoxylin and eosin staining revealed no ventricular enlargement at birth (two wild types, two asymptomatic heterozygotes and five homozygotes; Supplementary Fig. S4A), whereas mild enlargement was observed at 1.5 weeks of age (five asymptomatic heterozygotes and five homozygotes; Fig. 4A).

Because microglial activation occurs in several brain pathologies, including hydrocephalus [5, 15, 16], we also investigated changes in hippocampal microglial morphology at 1.5 weeks of age by examining coronal brain sections (40 μm Z stacks) immunostained with anti-Iba1 antibodies (three asymptomatic heterozygotes and three homozygotes) using confocal microscopy. The hippocampal microglia in *Hoatz*^*−/−*^ mice exhibited reduced process complexity compared with those in asymptomatic heterozygotes (Fig. 4B, C), consistent with an activated phenotype.

MRI performed when the mice were 3 weeks old (three wild-type and three homozygotes; a subset of the 12 mice that also underwent MRI at 6–10 weeks old, as described above) revealed that by 3 weeks of age, the degree of ventriculomegaly in *Hoatz*^*−/−*^ mice was comparable to that observed in adult mice (Supplementary Fig. S4B).

Transcriptomic analyses of the adult mouse hippocampus tissue were also conducted. Briefly, total RNA was extracted from freshly dissected hippocampi harvested from three age-matched male wild-type and *Hoatz*^*−/−*^ littermates (aged 15, 21, and 25 weeks) and then subjected to RNA sequencing. Principal component analysis revealed a clear separation between wild-type and null samples along the PC1 axis, accounting for 46.2% of the total variance (Fig. 4D).

Differential expression analysis, using a threshold of adjusted *p* < 0.05 and |log2 fold change| ≥2, revealed 41 upregulated genes, including *Trem2*, *Tyrobp*, *C1qa*, and *Lyz2*, and 953 downregulated genes in *Hoatz*^*−/−*^ mice compared with wild-type mice (Fig. 4E, F). Gene ontology and pathway enrichment analyses of the upregulated genes revealed significant overrepresentation of terms related to immune activation (leukocyte-mediated immunity and neutrophil degranulation), synaptic remodeling (synapse pruning and peptide ligand–binding receptors), and lipid mediator biosynthesis (icosanoid biosynthetic process) (Fig. 4G). Representative genes included *Trem2*, *Tyrobp*, *C1qa*, and *Lyz2* for immune activation; *Sst*, *Npy*, *Cort*, and *Ttr* for synaptic remodeling; and *Ltc4s*, *Cyp4f17*, and *Ptgds* for lipid signaling. Mitochondrial transcripts (*mt-Atp6* and *mt-Co2*) and *Ctss* were mapped to the prion disease pathway in the Kyoto Encyclopedia of Genes and Genomes, indicating alterations in metabolic and neurodegenerative pathways. Collectively, these transcriptomic profiles show that *Hoatz* deficiency is associated with neuroimmune activation and changes in synaptic and metabolic gene expression within the hippocampus, correlating with the observed ventriculomegaly phenotype.

Overall, these findings demonstrate the rapid emergence of ventricular expansion during early postnatal development, which is generally established by the time of weaning. The low incidence of abnormal deaths thereafter (Table 1) suggests that ventriculomegaly transitions into a relatively stable chronic phase, although the mechanisms underlying this stabilization remain unclear.

## Discussion

We characterized structural abnormalities in the brain of *Hoatz*^*−/−*^ mice using descriptive morphological analyses. Severe hydrocephalus was rare (Table 1), with the majority of *Hoatz*^*−/−*^ mice developing mild, reproducible ventriculomegaly (Figs. 1 and 2) that did not affect total brain size or volume. However, it caused considerable deformation of the surrounding parenchyma, mainly in the hippocampus (Fig. 3). Ventriculomegaly emerged acutely during the first 3 weeks after birth and then transitioned into a chronic phase, accompanied by changes in hippocampal microglial morphology and gene expression (Fig. 4).

The early postnatal onset of ventriculomegaly is consistent with the fact that *Hoatz* is a motile cilia and flagella-associated gene and that the ependyma undergoes motile ciliogenesis during the postnatal period [17, 18]. It remains unclear why a small subset of homozygotes (four females) developed severe hydrocephalus. Notably, Liu et al. reported a significant difference in CSF production rates in rodents according to sex, with young females producing ~30% more CSF than age-matched males [19]. This suggests that female brains may be more vulnerable to disturbances in CSF circulation arising from dysfunctional motile cilia.

The ventricular volume increased by ~4.7-fold in *Hoatz*^*−/−*^ mice, corresponding to an estimated 2.8-fold increase in ventricular surface area, assuming isotropic expansion (surface area ∝ volume^2/3^). Ependymal cells are terminally differentiated derivatives of radial glial cells that have fulfilled their neurogenic potential [20]. Thus, it is intriguing to consider the underlying mechanism of ventricular surface expansion. Possibilities include hypertrophy of individual ependymal cells, re-entry into the cell cycle, or recruitment of other glial cell types.

Recent large-scale human genetic studies have identified multiple genomic loci associated with hydrocephalus. Duy et al. identified *TRIM71* as a causative gene in prenatal hydrocephalus, demonstrating that *TRIM71* mutations cause premature neuroepithelial cell differentiation [21, 22]. Räsänen et al. reported that *SLCO1A2*, which encodes an organic anion transporter expressed in brain capillary endothelial cells [23], along with several additional loci, was associated with an increased risk of idiopathic normal pressure hydrocephalus (NPH) [24]. Notably, these human genetic studies did not identify genes associated with cilia and flagella motility, challenging the cilia hypothesis of hydrocephalus pathogenesis that has largely emerged from mouse mutant studies [25].

From an anatomical standpoint, this discrepancy may be due to the substantial size differences between human and mouse brains. In particular, the diameter of the cerebral aqueduct (the narrowest part of the ventricular system) is estimated to be roughly an order of magnitude larger in humans than in mice. According to the Hagen–Poiseuille law, which approximates the relationship between vessel diameter and pressure in laminar flow, resistance to CSF flow is inversely proportional to the fourth power of the aqueduct radius. Therefore, if the cerebral aqueduct in mice is ~ 10 times narrower than in humans, the resulting resistance would be ~10,000-fold greater. This steep increase in resistance may make mice far more vulnerable to disturbances in ependymal cilia motility, more readily leading to ventricular enlargement. In contrast, the substantially lower flow resistance in humans may result in similar degrees of ciliary dysfunction remaining subclinical, potentially explaining why motile cilia and flagella-associated genes are not prominent among human hydrocephalus risk loci. In line with this interpretation, Yoshida et al. simulated human CSF fluid dynamics, revealing that ciliary defects did not affect intracranial pressure but instead altered fluid exchange in the lateral ventricles [26].

However, several reports have identified cilia-related gene mutations in cases of human hydrocephalus [27–29], such as the findings of Yang et al., who reported mutations in cilia-related genes or genes highly expressed in ependymal cells in patients with NPH [30]. These observations raise the possibility that such genomic alterations impair ciliary motility in addition to other ependymal cell functions. Ependymal cells not only propel CSF via motile cilia but also contribute to water transport across the ventricular surface via aquaporin-4 [31]. In our study, some *Hoatz*^*−/−*^ mice exhibited edematous lacunar lesions in the corpus callosum and other periventricular white matter. Thus, *Hoatz* knockout may compromise both the structural integrity of motile cilia [6] and the epithelial barrier or transport properties of ependymal cells.

The ventriculomegaly observed in *Hoatz*^*−/−*^ mice, which develops during the early postnatal period but does not markedly affect overall brain size, suggests that any associated increase in intracranial pressure is likely to be moderate. In this respect, the mouse phenotype may share certain features with NPH in humans. Although intracranial pressure in *Hoatz*^*−/−*^ mice was not directly measured, this model may provide a complementary experimental framework for investigating the effects of moderately altered CSF dynamics. Notably, changes in microglial morphology and gene expression were observed in the hippocampus, consistent with an altered microglial state. Such changes may have the potential to influence neural circuit function, for example through cytokine-mediated modulation of inhibitory interneurons, including pathways involving interleukin-6 [32]. It remains unclear whether these neuroimmune alterations are specific to the *Hoatz* mutation or represent a more general response to hydrocephalus. Previous studies using a motile cilia–deficient *Ccdc39* mutant model of severe progressive neonatal hydrocephalus demonstrated that periventricular white matter injury is associated with inflammatory activation of resident microglia, and that pharmacological suppression of this response with bindarit improves myelination [33, 34]. These findings suggest that inflammatory activation of microglia may occur as a more general secondary consequence of disrupted CSF dynamics in hydrocephalus.

Recent human studies have reported immune alterations in idiopathic normal pressure hydrocephalus that may not be limited to the CNS but may also involve peripheral immune dysregulation [35, 36]. Given that cognitive impairment is a hallmark feature of NPH, it will be of interest to examine whether the hippocampal microglial changes observed in *Hoatz*^*−/−*^ mice are associated with functional alterations of the hippocampus.

This study has several limitations. Intracranial pressure and CSF flow were not directly measured, precluding definitive conclusions about the biomechanical and hydrodynamic basis of ventriculomegaly. Transcriptomic profiling relied on bulk hippocampal tissue with a limited sample size (*n* = 3 per genotype), reducing statistical power and preventing cell type–specific interpretation. All imaging and transcriptomic analyses were performed in males, whereas severe hydrocephalus occurred only in females, limiting assessment of sex-dependent effects. In addition, several supplementary experiments used heterozygous mice as controls; although heterozygotes were asymptomatic, this may affect comparability across experiments. Future work incorporating physiological measurements of intracranial pressure and CSF dynamics, single-cell or spatial transcriptomics, and sex-inclusive longitudinal analyses will be required to address these limitations.

Thus, future studies integrating intracranial pressure measurements with inflammatory profiling and hippocampal functional analyses are essential to elucidate how impaired CSF dynamics interact with neuroimmune pathways.

## Electronic supplementary material

Below is the link to the electronic supplementary material.

Supplementary Material 1

Supplementary Material 2

Supplementary Material 3

Supplementary Material 4

Supplementary Material 5

## Data Availability

The RNA sequencing data generated in this study have been deposited in the Gene Expression Omnibus (GEO) database under accession number GSE324472.
