# Supplementary figures and images for: Early-onset ventriculomegaly and neuroimmune alterations in Hoatz-deficient mice
Source: Fluids Barriers CNS. 2026 Apr 1;23:72. doi: 10.1186/s12987-026-00796-4 (PMC13169590; doi:10.1186/s12987-026-00796-4)

**A**

*Hoatz*<sup>-/-</sup> (10 weeks old)

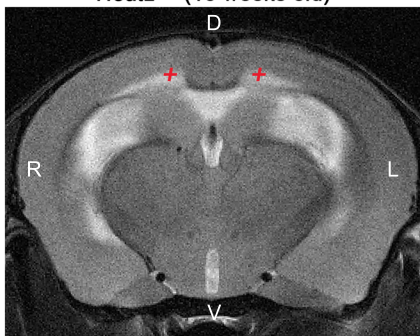

**B**

Coronal

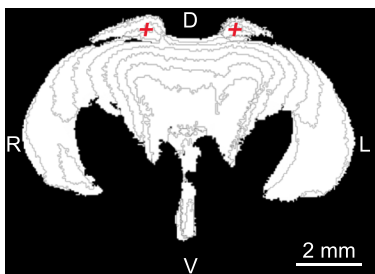

Horizontal

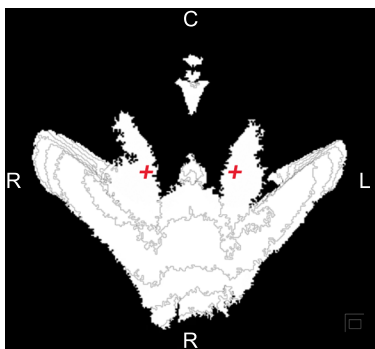

Sagittal

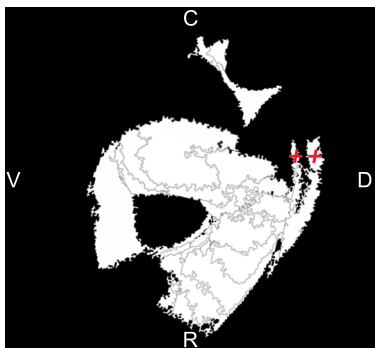

Supplement: Supplementary file 1 — Supplementary Material 1 [file 12987_2026_796_MOESM1_ESM.pdf]

*Hoatz*<sup>+/-</sup>

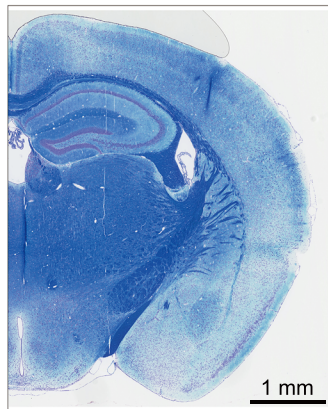

*Hoatz*<sup>+/-</sup>

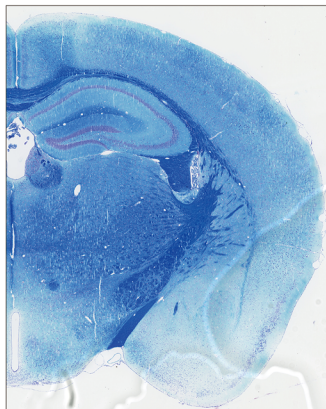

*Hoatz*<sup>-/-</sup>

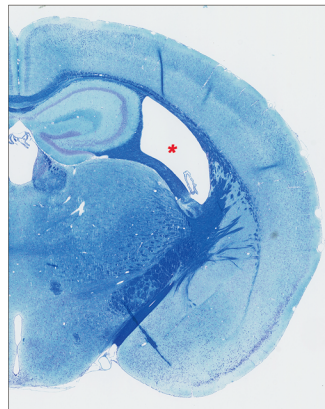

*Hoatz*<sup>-/-</sup>

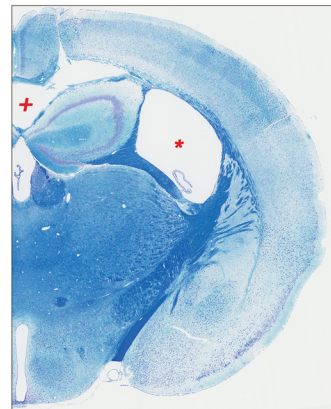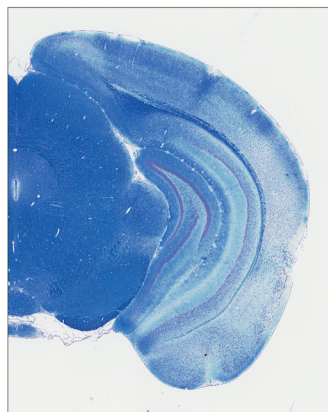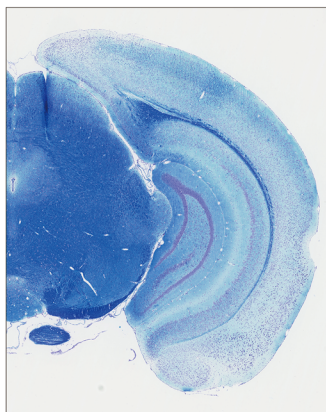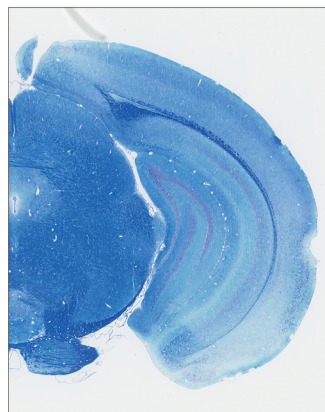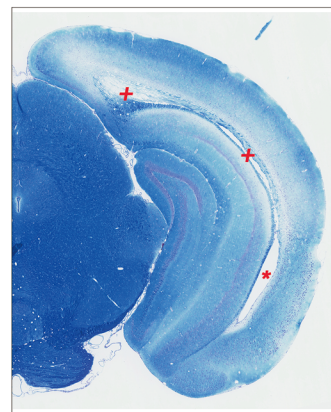

Supplement: Supplementary file 2 — Supplementary Material 2 [file 12987_2026_796_MOESM2_ESM.pdf]

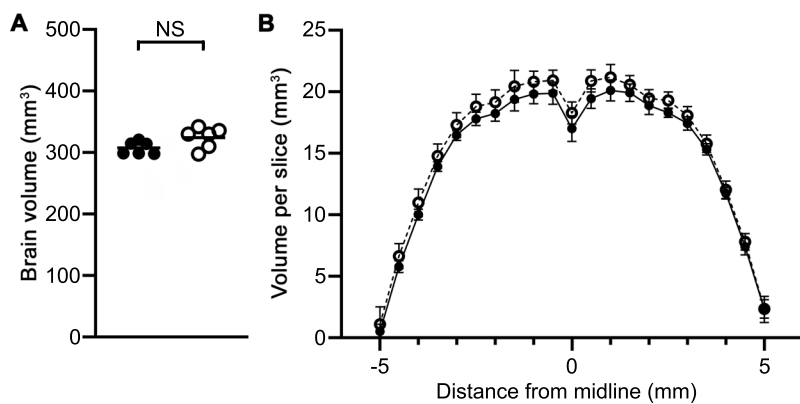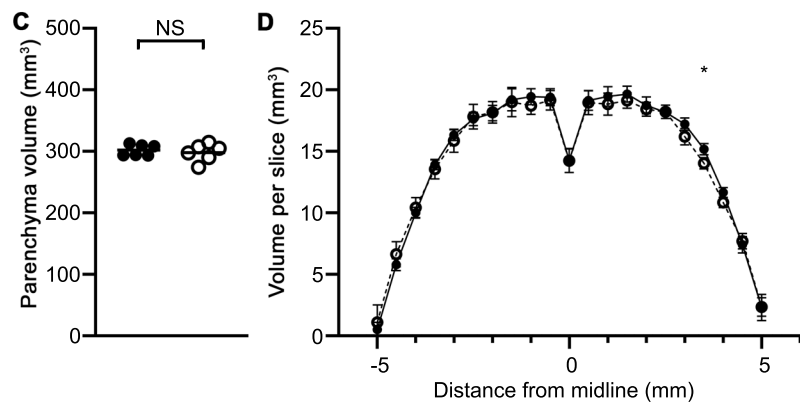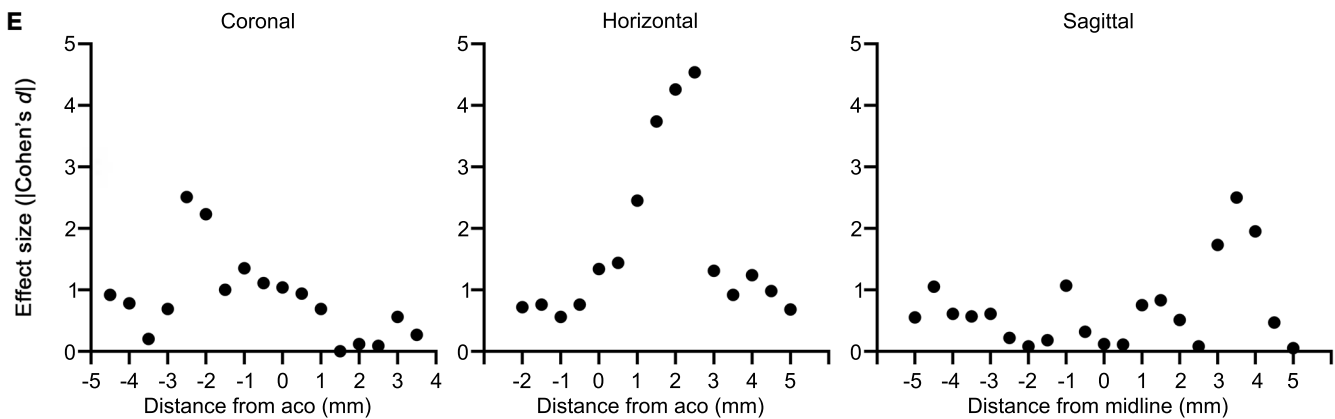

Supplement: Supplementary file 3 — Supplementary Material 3 [file 12987_2026_796_MOESM3_ESM.pdf]

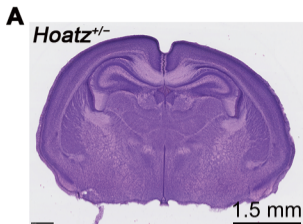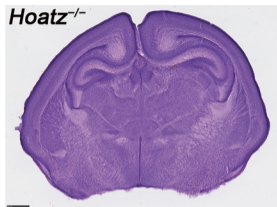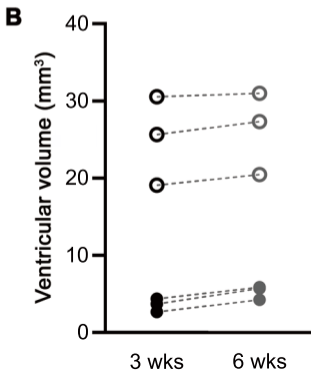

Supplement: Supplementary file 4 — Supplementary Material 4 [file 12987_2026_796_MOESM4_ESM.pdf]
