# Supplementary material for: Early-onset ventriculomegaly and neuroimmune alterations in Hoatz-deficient mice
Source: Fluids Barriers CNS. 2026 Apr 1;23:72. doi: 10.1186/s12987-026-00796-4 (PMC13169590; doi:10.1186/s12987-026-00796-4)
Supplement: Supplementary file 5 — Supplementary Material 5 [file 12987_2026_796_MOESM5_ESM.docx]

Figure Legends

**Figure S1.** Representative *Hoatz^−/−^* mouse exhibiting edematous lacunae in the corpus callosum (red crosses). (A) T_2_-weighted coronal MRI slice. (B) Segmentation of hyperintense regions from the same mouse, illustrating the spatial distribution of lacunae together with the enlarged ventricles. See Figure 2 and the main text for methodological details.

**Figure S2.** Representative Klüver–Barrera-stained coronal brain sections from formalin-fixed, paraffin-embedded adult male mice (two asymptomatic heterozygotes and two homozygotes). The upper and lower panels correspond to slices 70 and 86 of the Allen Brain Reference Atlas (adult mouse, coronal sections) [36], respectively. Enlarged lateral ventricles (red asterisks) and edematous lacunae (red crosses) were observed in homozygotes.

**Figure S3.** Brain and parenchymal volume measurements. (A–D) Filled circles, wild-type; open circles, null. (A) Comparison of total brain volume using sagittal sections (scatter dot plots with mean bars). (B) Slice-by-slice comparison of brain volume using sagittal sections (mean ± standard deviation). (C) Comparison of total parenchymal volume using sagittal sections. (D) Slice-by-slice comparison of parenchymal volume using sagittal sections. *, *P* = 0.0306 (Sidak multiple comparison test), n = 6. (E) Slice-by-slice comparison of effect sizes estimated using Cohen *d*.

**Figure S4.** Detection of ventriculomegaly in the early postnatal stage. (A) Representative hematoxylin and eosin-stained coronal brain sections on the day of birth. (B) Comparison of ventricular volume as determined by MRI at 3 and 6 weeks old in wild-type and homozygous mutant mice. For each animal, the corresponding MRI data acquired at 6 weeks of age (shown in Figure 2B) is displayed in gray and connected with dashed lines. Filled circles, wild-type; open circles, null.
